# *Fusobacterium nucleatum* and its associated systemic diseases: epidemiologic studies and possible mechanisms

**DOI:** 10.1080/20002297.2022.2145729

**Published:** 2022-11-14

**Authors:** Zixin Fan, Pengzhou Tang, Cheng Li, Qi Yang, Yan Xu, Chuan Su, Lu Li

**Affiliations:** a Department of Periodontics, Affiliated Stomatological Hospital of Nanjing Medical University, Nanjing, Jiangsu, China; b Jiangsu Key Laboratory of Oral Disease, Nanjing Medical University, Shanghai road 1, Nanjing, Jiangsu, China; c Jiangsu Province Engineering Research Center of Stomatological Translational Medicine, Nanjing Medical University, Nanjing, Jiangsu, China; d State KeyLaboratory of Reproductive Medicine, Jiangsu Key Laboratory of Pathogen Biology, Department of Pathogen Biology and Immunology, Center for Global Health, Nanjing Medical University, Nanjing, Jiangsu, China

**Keywords:** *Fusobacterium nucleatum*, systemic diseases, periodontal disease, FadA, microbiome

## Abstract

**Background:**

*Fusobacterium nucleatum* (*F. nucleatum*) is an anaerobic oral commensal and the major coaggregation bridge organism linking early and late colonisers. In recent years, a large number of studies suggest that *F. nucleatum* is closely related to the development of various systemic diseases, such as cardiovascular diseases, adverse pregnancy outcomes, inflammatory bowel diseases, cancer, Alzheimer's disease, respiratory infection, rheumatoid arthritis, etc.

**Objective:**

To review the effect of *F. nucleatum* on systemic diseases and its possible pathogenesis and to open new avenues for prevention and treatment of *F. nucleatum*-associated systemic diseases.

**Design:**

The research included every article published up to July 2022 featuring the keywords 'Systemic diseases' OR 'Atherosclerotic cardiovascular diseases' OR 'Atherosclerosis' OR 'Adverse pregnancy outcomes' OR 'Inflammatory bowel disease' OR 'Ulcerative colitis' OR 'Crohn’s disease' OR 'Cancers' OR 'Oral squamous cell carcinomas' OR 'Gastrointestinal cancers' OR 'Colorectal cancer' OR 'Breast cancer' OR 'Genitourinary cancers' OR 'Alzheimer’s disease ' OR 'Rheumatoid arthritis' OR 'Respiratory diseases' AND '*Fusobacterium nucleatum*' OR 'Periodontal pathogen' OR 'Oral microbiota' OR '*Porphyromonas gingivalis*' and was conducted in the major medical databases.

**Results:**

*F. nucleatum* can induce immune response and inflammation in the body through direct or indirect pathways, and thus affect the occurrence and development of systemic diseases. Only by continuing to investigate the pathogenic lifestyles of *F. nucleatum* will we discover the divergent pathways that may be leveraged for diagnostic, preventive and therapeutic purposes.

## Introduction

Periodontal disease (PD), one of the most common inflammatory diseases in adults, comprises a wide range of inflammatory conditions that jeopardize the supporting structures of the teeth (the gingiva, bone and periodontal ligament). PD is initiated by bacterial biofilm, which interacts with the host immune defense system, further aggravating the inflammatory response [1]. Recently, mounting evidence has supported PD as a potential risk factor for multiple systemic diseases [2–6]. These diseases include cardiovascular diseases, adverse pregnancy outcomes, gastrointestinal and colorectal cancer, Alzheimer’s disease, respiratory infection, rheumatoid arthritis, etc. In addition, studies show that periodontal pathogens can spread to different parts of the body through direct dissemination, blood transmission, immunization and other ways, causing systemic or local infection, thus exerting influence on the occurrence and development of systemic diseases [7–9]. *F. nucleatum*, a Gram-negative anaerobe, is one of the most abundant species in the oral cavity for both diseased and healthy individuals [6]. It is a coaggregation bridge organism, which links primary and late colonisers by coaggregation-mediated mechanisms and by promoting growth of other anaerobes [10,11]. Its virulence mechanism involves colonization, invasion, as well as induction of aberrant inflammation and tumorigenesis [12]. In the past decade, *F. nucleatum* has become a hot research topic because of its increasingly revealed associations with extraoral diseases. The surface adhesin FadA expressed by *F. nucleatum* could increase permeability and promote *F. nucleatum* penetration of endothelial cells by binding to vascular endothelial cadherin (VE-cadherin) [13–15]. This phenomenon also supported the opinion that hematogenous transmission might be one route used by bacteria to spread from the oral cavity to deeper organs with crossing of the endothelial barrier as a key step in the process [16]. In this review, we delve into recent discoveries and prospects of *F. nucleatum*-related researches, including our evolving understanding of its mechanistic role in promoting systemic diseases and the challenges of developing diagnostic and therapeutic methods for *F. nucleatum*-associated systemic diseases.

## Association between *F. nucleatum* and systemic diseases

### Atherosclerotic cardiovascular diseases (ACVDs)

ACVDs, including coronary artery disease and stroke, are one of the most common causes of death in the elderly [17]. Atherosclerosis (AS) is the pathological basis of ACVDs, and the widely recognized risk factors of AS include hyperlipidemia, hypertension, smoking, diabetes, obesity, immune damage and genetic factors. However, studies have shown that up to half of AS patients may not be under those risks [18]. In recent years, researchers have found that oral bacteria play a role in the occurrence and development of AS. Oral bacterial DNA was initially detected in human atherosclerotic plaques by Haraszthy *et al*. [19], as well as in coronary artery biopsies from patients with coronary artery disease and endarterectomy specimens from patients undergoing surgical treatment for atherosclerosis by Ford *et al*. [20,21]. Some of these bacteria are well-known periodontal pathogens, such as *Aggregatibacter actinomycetecomitans* (*A. actinomycetecomitans), Porphyromonas gingivalis* (*P. gingivalis), Tannerella forsythia* (*T. forsythia), Prevotella intermedia* (*P. intermedia), F. nucleatum, Campylobacter rectus*, and *Treponema denticola* (*T. denticola*) [19–21], suggesting that PD may be associated with AS. Recent meta-analyses also highlighted the close correlation between PD and AS [22,23]. Moreover, the treatment of PD was also proved to have a certain inhibitory effect on progression of ACVDs independent of traditional ACVDs risk factor management [24].

*F. nucleatum* is one of the oral bacteria detected in the atherosclerotic plaques [21,25,26] and the frequency of detected *F. nucleatum* in atherosclerotic plaques and blood vessels is directly related to the severity of PD [27]. In 31 carotid arterectomy specimens, the detection rate of *F. nucleatum* was 34% [21]. Subsequent animal experiments proved that infection of *F. nucleatum* alone did not promote AS [28], but in the multi-bacterial infection model, *F. nucleatum* would act synergistically with other organisms in the development of AS [29,30]. There are several etiological hypotheses for AS, all of which can be attributed to the ‘injury response’ [31]. During the formation of AS, serum lipoprotein concentration, endothelial permeability, and binding of lipoprotein to intima are considered to be three important pathogenic factors [32]. Permeability of endothelial cells is a key factor in the pathogenesis of ACVDs, because the formation of AS requires not only monocytes and lipoprotein to penetrate the endothelium, but also lipoprotein to accumulate in intima [33].

Firstly, *F. nucleatum* can promote the progression of AS by affecting endothelial cell permeability through various mechanisms. *F. nucleatum* possesses a best-characterized surface adhesion called FadA [34]. FadA has two forms: one is an undamaged pre-FadA consisting of 129 amino acids and the other is a secreted mature FadA (mFadA) consisting of 111 aa residues [14]. Pre-FadA and mFadA form a high molecular weight complex, FadAc, which can bind to VE-cadherin on endothelial cells, causing the latter to migrate from cell–cell junctions to intracellular compartments [15]. This results in an endothelium so permeable that even passage of bacteria is allowed, a likely reason why *F. nucleatum* is often found in mixed infections at extra-oral sites. In addition, *F. nucleatum* challenge markedly impaired cell proliferation and apoptosis in endothelial cells, destroying the original equilibrium state and leading to endothelial damage [35]. *F. nucleatum* infection was also observed to affect the expression of endothelial cell surface markers and modulate receptors for VEGF on endothelial cells, resulting in impaired tissue vascularization during inflammation [36]. On the whole, after entering the bloodstream, *F. nucleatum* further impairs vascular endothelial integrity by inhibiting endothelial cell proliferation, destroying endothelial cell structure and function, and inhibiting the vascularization of damaged tissues during inflammation, thus paving the way for the invasion of other bacteria [34–36].

Secondly, many studies have reported that human heat shock proteins (hHSPs) are closely related to AS. Antibodies directed against bacterial GroEL cross-react with hHSP60 on endothelial cells may result in endothelial dysfunction and the subsequent development of AS [37]. Lee *et al*. proved that the heat-shock protein GroEL of *F. nucleatum* stimulated atherosclerotic risk factors through several mechanisms [38]. *F. nucleatum* GroEL upregulated the expression of chemokines, such as interleukin-8 (IL-8) and monocyte chemoattractant protein-1 (MCP-1), and cell adhesion molecules, such as vascular cell adhesion molecule-1 (VCAM-1), intercellular adhesion molecule-1 (ICAM-1), and E-selectin. This is an important step in the pathogenesis of endothelial dysfunction. In addition, GroEL could increase procoagulant activity by upregulating tissue factor (TF) and downregulating TF pathway inhibitor (TFPI) in endothelial cells. This prothrombotic response may be associated with plaque progression and instability. GroEL also induced monocyte adhesion to and transmigration through endothelial cells in concert with increased uptake of lipids in atherosclerotic lesions, and promoted monocyte differentiation into pro-inflammatory macrophages and, eventually, foam cells.

Thirdly, chronic oral infection with *F. nucleatum* as monoinfection alone does not promote the induction of AS, but may conversely inhibit plaque formation [28]. In this research, 24-week *F. nucleatum*-infected mice developed minimal aortic plaque, which was significantly smaller than sham-infected mice, and fewer F4/80^+^ macrophages were detected in the intimal layer of 24-week-infected mice than 12-week-infected mice. Additionally, there was no increase in macrophage infiltration or T-cell infiltration into the inner and outer membrane layers in infected mice at 24 weeks, suggesting that chronic *F. nucleatum* infection reduces inflammation in the aorta, which may in part explain the minimal development of atherosclerotic plaques. Although all lipid fractions were statistically elevated in *F. nucleatum*-infected mice, serum NO was not altered. Consistent with the minimal plaque observed in 24-week-infected mice, the result demonstrated no vascular endothelial dysfunction in animal model.

Some scholars used the multi-bacterial infection models to show that the inclusion of *F. nucleatum* promoted the endothelial dysfunction and AS plaque progression by upregulating expression of different aortic Toll-like receptors (TLRs) and inflammasome signal transduction [29,30]. Subsequent studies further demonstrated that *F. nucleatum* up-regulated pro-inflammatory factors such as IL-1α, IL-6 and TNF-α through activation of TLR-MyD88-NF -κB in endothelial cells, resulting in the upregulation of ICAM, VCAM and MCP-1 [35].

Due to the interdependence (physical, metabolic, and nutritional) of periodontal bacteria, no single periodontal bacterial species is effective in inducing aortic disease pathology, for which a multimicrobial consortium is responsible. In summary, more researches are needed to confirm these arguments that *F. nucleatum* may not be involved in AS alone, but rather act synergistically with other organisms, in particular by disrupting cell–cell junctions, breaking down endothelial integrity and leading to invasion [28–31].

**Figure 1.**
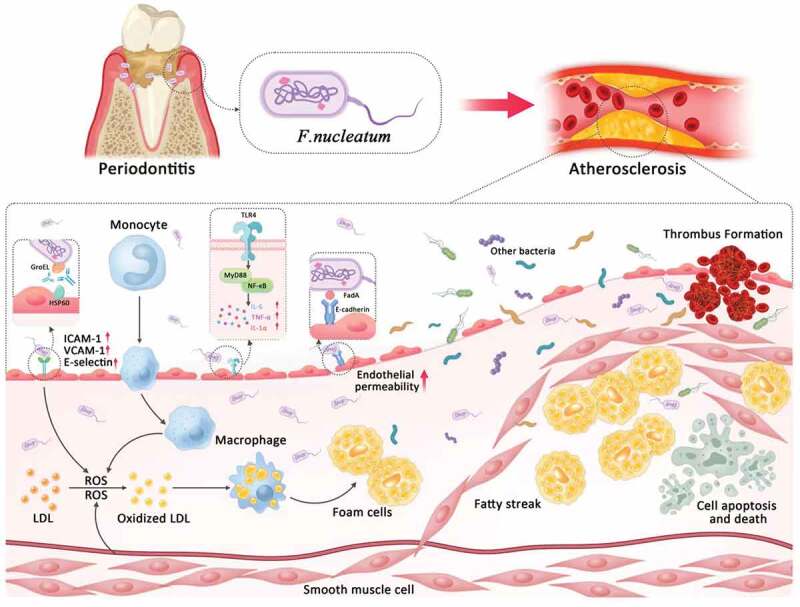
The possible mechanisms by which *F. nucleatum* contributes to atherosclerosis. HSP60, heat shock protein-60; ICAM-1, intercellular adhesion molecule-1; VCAM-1, vascular cell adhesion molecule-1; LDL, low-density lipoprotein; ROS, reactive oxygen species; IL-6, interleukin-6; TNF-α, tumor necrosis factor-α.

Furthermore, we found that both *P. gingivalis* and *F. nucleatum in vitro* can induce the expression of fatty acid-binding protein 4 (FABP4) mRNA and protein depending on the JNK/AP-1 pathway, causing an increase of lipid uptake and foam cell transformation in macrophages [39]. Human studies showed that serum levels of antibodies against *P. gingivalis* correlated with serum levels of FABP4 in humans, whereas no association occurred between *F. nucleatum* antibody titers and FABP4 levels, which might indicate that *P. gingivalis* is superior to *F. nucleatum* in inducing FABP4 in humans. Further study is needed to define the reason underlying the difference in cell-based models and human studies. The mechanisms between *F. nucleatum* and AS are summarized in Figure 1.

### Adverse pregnancy outcomes (APOs)

APO is a broad term including preterm labor, chorioamnionitis, preterm premature rupture of membranes, preeclampsia, miscarriage, intrauterine growth retardation, low birth weight, stillbirth, neonatal sepsis, etc.

*F. nucleatum* is one of the most prevalent species and by far the most prevalent oral species implicated in APOs and has been found to be significantly enriched in a wide variety of placental and fetal tissues including amniotic fluid, fetal membranes, cord blood, neonatal gastric aspirates, fetal lung and stomach, associated with preterm birth, preterm premature rupture of membranes, chorioamnionitis, early-onset neonatal sepsis, stillbirth and preeclampsia, either as the sole infectious agent or in mixed infections [40].

The strains of *F. nucleatum* identified in amniotic fluid and placenta appear to match those from the maternal or the partner subgingival sites, providing the human evidences that the bacteria originated from the mother’s subgingival plaque and translocated to the placenta and fetus, causing acute inflammation that eventually leads to APOs [41]. Animal studies also show that injecting saliva or subgingival plaque samples into mice leads to infection of the murine placenta with oral commensal species, including *F. nucleatum*, demonstrating that the oral bacteria are capable of translocation to the fetal-placental unit [16]. Concurrent detection of *F. nucleatum* in amniotic fluid and cord blood associated with preterm birth indicates its ability to spread to different placental and fetal compartments [16].

It has been assumed that *F. nucleatum* translocates from the maternal oral cavity to the intrauterine cavity via hematogenous transmission [42–44]. This hypothesis is supported by results from animal studies [45,46]. In a pregnant mouse model, *F. nucleatum* was injected into the tail vein of pregnant mice to mimic dental bacteremia, which led to bacterial colonization in the decidua of the mouse placenta, followed by spread to the fetal membranes, mimicking chorioamnionitis in humans [45]. A recent case-cohort design based on placental specimens from 320 subjects showed that the human placenta harbors a low abundant microbiome closely mimicking the human oral microbiome, further supporting the hypothesis of blood-borne transmission [47].

Periodontal pathogens can cause adverse pregnancy outcomes through two major mechanisms identified in the consensus report from the joint European Federation of Periodontology/American Academy of Periodontology workshop on periodontitis and systematic diseases: one is that periodontal pathogens directly enter the blood circulation system, or an ascending route via the genitourinary tract, thus invading the fetal-placenta unit; the other is that pro-inflammatory cytokines, such as IL-6, IL-8, and TNF-α, which are locally produced in periodontal tissues, enter the circulatory system, directly exerting negative influence on the fetal-placenta unit, or circulate to the liver and increase the systemic inflammatory state through the acute phase of protein reactions, which in turn affects the fetal placental unit [48,49]. In both ways, periodontal pathogens may cause inflammation in the placental tissues of pregnant women, increase levels of prostaglandin E2 and TNF-α in the amniotic fluid, and contribute to the onset of APOs [50].

Several adhesin molecules, including Fap2 and FadA, have been identified for colonization of the *F. nucleatum* in the placenta of mice [13,51–53]. Among them, FadA is the best characterized and plays a critical role in the murine model of infection [15]. Binding of *F. nucleatum* FadA to VE-cadherin not only increases the endothelial permeability, but also allows *F. nucleatum* and other oral bacteria to disseminate into and from the circulation. This may explain why *F. nucleatum* is frequently detected concurrently with other oral species in intrauterine infections in humans [6]. *In vivo*, an *F. nucleatum* mutant without FadA is significantly defective in placental colonization, while its complement clone restores the colonization [45,54].

More studies reported that colonization of *F. nucleatum* in the mouse placenta led to preterm and/or term fetal death, occurring 2–3 days following bacterial injection, accompanied by placental neutrophil infiltration, similar to that observed in humans [45]. Although *F. nucleatum* activates both TLR2 and TLR4 *in vitro* and in murine placentas, it induces inflammatory responses via TLR4, accompanied by neutrophil infiltration into the decidua [46]. In mice lacking TLR4, or in wild-type mice treated with a TLR4 antagonist, *F. nucleatum* colonizes the placenta to a similar extent as in untreated wild-type mice without eliciting inflammatory responses, resulting in a reduced fetal death rate, which suggests that inflammation rather than the bacteria per se is the cause of fetal demise. In contrast, the effect of TLR2 is insignificant because there is no change in fetal loss or inflammatory response in TLR2-knockout mice compared to wild-type mice.

Scholars have confirmed that inflammatory biomarkers in the maternal serum may include IL-6,C-X-C motif chemokine 8 (CXCL8) and CC chemokine ligand 2 (CCL2) [55–59]. Receptor-interacting protein kinase 2 (Ripk2) may contribute to *F. nucleatum*-induced production of IL-6 by activating NF-κB signaling in murine macrophages and human decidual stromal cells (hDSCs) [60]. Ripk2 also contributed to inducible nitric oxide synthase (iNOS) gene expression and NO production in macrophages and promoted the production of CXCL8 and CCL2, which was reduced by Ripk2 inhibitors. These results suggested that *F. nucleatum* infection results in APOs by inducing aberrant production of cytokines and chemokines through NOD1/NOD2-Ripk2-mediated signaling. In conclusion, although it would be beneficial to regulate Ripk2 signaling to prevent APOs caused by bacterial infections, further studies using animal models are needed to elucidate whether Ripk2 is involved in *F. nucleatum*-induced APOs and Ripk2 inhibitors are beneficial to prevent the occurrence of APOs.

### Inflammatory bowel disease (IBD)

IBD, a heterogeneous set of inflammatory disorders of the gastrointestinal (GI) tract and a global public health issue of increasing importance, presents as two major clinical phenotypes: ulcerative colitis (UC) and Crohn’s disease (CD). UC is a continuous inflammation of the colonic mucosa and submucosa, usually involving the rectum initially, then gradually spreading to the whole colon, whereas CD is usually transmural and can affect any area of the gastrointestinal tract, which is a discontinuous full-layer inflammation, most commonly involving the terminal ileum, colon, and perianal [61]. The etiology of IBD is still not completely understood. Yet, several studies have supported the hypothesis that its onset is due to a convergence of host genetic factors and environmental triggers resulting in changes of the host immune response to intestinal microbes [62,63]. Therefore, the role of intestinal microbiota alteration is repeatedly discussed in literatures [64].

Back in 2011, Strauss *et al*. have reported that *F. nucleatum* isolated from inflamed biopsy tissue from IBD patients is significantly more common and invasive than from healthy controls [65]. It has been reported that *F. nucleatum* is obviously enriched in feces of IBD patients and its abundance has a positive correlation with patients’ disease activity, and administration of *F. nucleatum* markedly exacerbates colitis in DSS mice model [66]. This confirms the findings of previous clinical studies [67–69]. In addition, scholars found that patients with IBD have a significantly increased risk of PD, and exhibit more severe periodontal symptoms when oral hygiene conditions are similar [70–72]. Recent studies also have suggested a bidirectional association between IBD and PD based on statistics of the prevalence and clinical manifestations of both diseases, whereas the microbial etiological correlation and common risk factors of the two diseases remained unclear [72–74].

Kitamoto *et al*. discussed that the mode of the relocation of oral bacteria from the oral cavity to the gut mucosa may include hematogenous route, enteral route and other possible factors [75]. First, oral mechanical injuries and dental procedures enable oral bacteria to spread into the systemic circulation hematogenously, and inflammatory conditions of the oral cavity, namely periodontitis, may facilitate bacteremia [76,77]. In addition, oral bacteria are known to invade and survive inside immune cells, such as dendritic cells and macrophages, indicating that oral bacteria may hijack host immune cells to serve as Trojan horses for dissemination from oral mucosa to gut mucosa [50]. Second, enteral spreading is a plausible route worth paying attention to. The colonization resistance by the gut resident microbiota is considered to be the major barrier that prevents the gut colonization of swallowed oral bacteria [75,78]. Meanwhile, the majority of oral resident bacteria are so sensitive to the gastric acid that ingested oral bacteria will be sharply reduced when they are passing the stomach [75,79,80]. Therefore, when dysfunction of gastric barrier or/and disruption of gut colonization resistance happens, there will be a significant increase in the ectopic gut colonization by oral bacteria. Other possible factors include immune depression and poor oral health [81,82]. Yet further studies are needed to clarify the transmission methods to the gut mucosa by oral bacteria.

In this review, we only discuss the possible pathogenesis of *F. nucleatum* in IBD. Firstly, *F. nucleatum* could damage the epithelial barrier integrity and increase permeability by downregulating the expression of the tight junction proteins zonula occludens-1 (ZO-1) and occludin, which are markers of intestinal mucosal barrier function [66]. Additionally, *F. nucleatum*-mediated mucosal barrier damage could be promoted by targeting caspase activation and recruitment domain 3 (CARD3) which in term activated the endoplasmic reticulum stress (ERS) pathway [83]. Secondly, *F. nucleatum* could regulate M1 macrophage skewing to induce colitis [84]. Liu *et al*. also reported that *F. nucleatum* could exacerbate intestinal inflammation through upregulating cytokine secretion, such as IL-1β, IL-6, and IL-17, activating STAT3 signaling pathway, enhancing proliferation of CD4^+^ T cell and differentiation to Th1 and Th17 [66]. Li *et al*. found that the presence of *F. nucleatum* and FadA gene increased in UC patients, especially in patients with severe colitis and pancolitis, suggesting that FadA may play a significant role in the pathogenesis of UC [85]. Nevertheless, the exact mechanism of this association is not fully understood, more studies are required to better elucidate the role of *F. nucleatum* and FadA gene in UC.

Using ApoE^−/−^ mice model, Yan *et al*. reported that non-surgical periodontal treatment triggered modulation of gut microbiota, facilitating recovery to a healthy microbiome situation [86]. It also strengthened the intestinal mucosal barrier which was impaired by periodontitis, resulting in a stronger nonspecific immune function. Based on this paper and the above relationship between *F. nucleatum* and IBD, we reasonably assume that *F. nucleatum* abundance maybe indicative of development of IBD. Moreover, targeting *F. nucleatum* may be effective to shorten disease course and prevent the development of IBD. Since there is no explicit cure for IBD, understanding the intrinsic mechanism of *F. nucleatum* may provide a new insight in optimizing current therapeutic strategies.

In addition, IBD has been recognized as a risk factor for colorectal cancer (CRC), the infection of *F. nucleatum* in intestinal tract may be the common pathogenesis of these two diseases [87]. The connection between *F. nucleatum* and CRC will be discussed in detail later.

### Cancers

#### Oral squamous cell carcinomas (OSCC)

OSCC is a malignant tumor occurring in the oral epithelium, which is the main type of head and neck squamous cell carcinoma (HNSCC). Al-Hebshi,*et al*. firstly showed that *F. nucleatum* is associated with OSCC from an epidemiological perspective [88]. Subsequently, scholars used 16S rRNA to analyze the microbiome within healthy normal and tumorous (primary and metastatic) human tissues from the oral cavity, larynx-pharynx, and lymph nodes [89]. The microbiota associated with tumors supported altered abundances in the phyla *Fusobacteria, Firmicutes, Actinobacteria* and *Proteobacteria*. Most notably, a significant reduction in the abundance of *Streptococcus* species and an increase in the abundance of *Fusobacterium* species were observed in both primary and metastatic samples. Resphera Insight applied to saliva samples from HNSCC patients and healthy controls led to the first discovery that *F. nucleatum* enriched in a subset of saliva samples from HNSCC patients, when compared with controls [90]. Additionally, many species of anaerobic bacteria have been proposed to be involved in carcinogenesis [91]. Nagy et al. detected significantly larger quantities of *Porphyromonas* and *Fusobacterium* species in OSCC tissue samples compared to samples from healthy mucosa [92]. *F. nucleatum* is closely associated with the development of oral cancer by several mechanisms.

Firstly, when *F. nucleatum* invades gingival epithelial cells, NF-κB and NOD-like receptor 3 (NLRP3) are simultaneously activated [93]. NF-κB would subsequently translocate to the nucleus where it stimulates expression of pro-IL-1β gene. NLRP3 inflammasome would induce autocatalytic activation of caspase 1, resulting in release of IL-1β, one of the most important proinflammatory cytokines associated with cancer pathogenesis [94]. Furthermore, once caspase 1 is activated, other danger-associated molecular patterns (DAMPs) (also known as danger signals) will be released, such as highmobility group box 1 protein (HMGB1) and apoptosis-associated speck-like protein (ASC), which further amplify immune response [93]. The inflammasome/IL-1β pathway has been reported to be involved in HNSCC progression [94,95]. Aral *et al*. further verified that *F. nucleatum* could promote IL-1β by increasing AIM2 and downregulating POP1 in HNSCC *in vitro* [96]. *P. gingivalis* and adenosine triphosphate (ATP) with or without *F. nucleatum* upregulated NLRP3, IL-1β by downregulating POP1. Moreover, P. gingivalis and F. nucleatum can initiate the overexpressed NLRP3, activate upstream signal molecules of ataxia-telangiectasia and Rad3 related (ATR)-checkpoint kinase 1 (CHK1), promoting the growth and proliferation of oral cancers [97].

Secondly, *F. nucleatum* induces activation of protein kinase p38 in infected cells, promoting the secretion of matrix metalloproteinase-13 (MMP-13) and MMP-9, which contributes to tumor invasiveness [98].

Thirdly, DNA damage plays a significant role in the development and progression of oral cancer. It has been reported that the Ku70/p53 signaling pathway may be involved in the excessive proliferation of *F. nucleatum*-infected OSCC cells due to DNA damage [99]. If Ku70 protein levels are too low to repair severely damaged DNA, OSCC cells will proliferate abnormally [100,101]. Nevertheless, the interplay between *F. nucleatum* and Ku70 is yet to be explained.

In addition, the levels of tumor suppressor protein p27, a member of the cyclin-dependent kinase inhibitor (CDK) family, would be downregulated in *F. nucleatum*-infected cells, which leads to cell-cycle arrest in the S phase and to increased cell proliferation [100]. This process is correlated with adverse cancer prognosis.

Moreover, infection of oral epithelial cells with *F. nucleatum* was found to contribute to the induction of epithelial–mesenchymal transition (EMT) through lncRNA MIR4435-2HG/miR-296-5p/Akt2/SNAI1 pathway [102]. EMT is an important biological process through which epithelial-derived malignant tumor cells acquire the ability to migrate and invade [103].

#### Gastrointestinal (GI) cancers

GI cancers are in the forefront of all malignant tumor in morbidity and mortality, including esophageal, gastric, colorectal, liver, and pancreatic cancer. A case-control study examined the salivary microbiota in patients with GI cancers and evaluated their differential distribution based on the cancer sites, suggesting the bacterial diversity and composition of saliva are related to GI cancers [104].

It was reported that *F. nucleatum* can not only promote the progression of GI tumor, but also contribute to the chemo-resistance of GI cancer [105]. We have summarized recent progress in the pathogenesis of *F. nucleatum-*related GI cancers.

##### *Colorectal cancer (CRC*)

CRC is one of the most common malignant tumors. Its morbidity and mortality rank among the top three cancers in the world, and are increasing in recent years [106]. Recent studies have revealed an enrichment of *Fusobacterium* species in human CRCs and adenomas compared with adjacent normal tissue [87,107–110], and the most abundant species is *F. nucleatum*, suggesting that *F. nucleatum* is involved in the development of CRC. Castellarin *et al*. verified overabundance of *F. nucleatum* in colorectal tumor specimens versus matched normal control tissue by quantitative PCR analysis and observed that *F. nucleatum* was positively associated with lymph node metastasis [87]. Subsequent studies implied that *F. nucleatum* can be used as a risk biomarker of CRC [111–114]. Increased levels of *F. nucleatum* correlated with the genetic and epigenetic aberrancies in CRC, such as the CpG island methylator phenotype (CIMP), microsatellite instability (MSI), and MLH1 methylation [108,115, 116–118].

*F. nucleatum* can alter the composition of the residual microbiota, with a consortium of inflammatory responses, virulence factors and impaired epithelial signaling in the context of a polymicrobial oral biofilm with synergistic properties, resulting in intestinal dysbiosis, contributing to the initiation and progression of CRC [118].

In respect to association between *F. nucleatum* and CRC oncogenesis, two pathways must be mentioned. One of the pathways is the virulence factor FadA of *F. nucleatum* which would activate the β-catenin signaling pathway when present on the VE-cadherin, initiating inflammatory responses, thus boosting the genes of the transcription factors NF-κB, pro-inflammatory cytokines such as IL-6, IL-8, and IL-18, Wnt7a, Wnt7b, Wnt9a, transcription factors lymphoid enhancer factor (LEF) /T cell factor (TCF), Myc, and Cyclin D1, and the parts of the Wnt pathway [119]. *F. nucleatum* also can activate β-catenin signaling through TLR4/P-PAK1 cascade [120]. In pathway two, the Fap2 of *F. nucleatum* mediated its enrichment in CRC by binding to tumor-overexpressed Gal-GalNAc, further intensifying the inflammatory response [121]. In pathway three, *F. nucleatum* lipopolysaccharide (LPS) could activate TLR4/MyD88/NF-κB, inducing high expression of miR21; the elevated miR21 could reduce levels of RASA1, while the RASA1 could active MAPK pathway [122. Meanwhile, *F. nucleatum* was found to inhibit the expression of miR-18* and miR-4802 through TLR4/MyD88 immune signals [123]. The loss of these miRs avoided chemotherapy-induced apoptosis by activating autophagy in CRC cells. In addition, when *F. nucleatum* invades into CRC cells, reactive oxygen species (ROS) could be induced, which subsequently lead to DNA damage [124,125].

The above findings bring us to the next question: how *F. nucleatum* modulates tumor immune microenvironment in CRC? On the one hand, *F. nucleatum* could selectively recruit tumor-infiltrating myeloid cells and promotes tumor progression [126]. On the other hand, Fap2 of *F. nucleatum* could interact with TIGIT, an immunoregulatory signaling receptor on NK and T cells, and this Fap2-TIGIT interaction could reduce killing of tumor cells by NK and tumor-infiltrating lymphocytes and inducing lymphocyte apoptosis [127–129].

Due to the biological characteristics of *F. nucleatum* and its relationship with CRC, studies on *F. nucleatum* are increasing gradually, but our understanding of *F. nucleatum* is still insufficient at present. Further researches are needed on how *F. nucleatum* can be a therapeutic target to reduce the risk of CRC. The researchers found that detection of fecal abundance of *F. nucleatum* can be used as a rapid and non-invasive diagnostic technique for CRC screening [112]. Combined with fecal occult blood test, the positive rate and accuracy of CRC diagnosis can be improved. In addition, serum IgA or IgG antibodies against *F. nucleatum* also have potential diagnostic value [130]. Scholars found that a ‘robust’ diet rich in whole grains and fiber could effectively reduce the individual’s risk of developing *F. nucleatum-*associated CRC, but the risk of CRC did not seem to change in patients lacking *F. nucleatum* [131]. Moreover, metronidazole treatment can significantly reduce tumor volume in a xenograft model of CRC enriched with *F. nucleatum* [132]. However, metronidazole broadly targets anaerobic bacteria. Therefore, it is important to search for narrow-spectrum antibiotics that are specific to *F. nucleatum* and only targeted at tumor tissue.

##### Other gastrointestinal (GI) cancers

Yamamura *et al*. reported that *F. nucleatum* enrichment was associated with a worse prognosis in esophageal cancer by activating chemokines, such as CCL20 [133]. In a study by Mitsuhashi *et al*., the detection rate of *Fusobacterium* spp. in pancreatic cancer tissue specimens was 8.8% [134], while *F. nucleatum* was not detected in pancreatic cancer tissue in another study by Yamamura *et al*. [135]. Castan˜o-Rodrı´guez *et al*. found that several bacterial groups, including *Lactococcus, Veilonella, Fusobacterium*, and *Leptotrichia* species were enriched in gastric cancer [136]. Heish *et al*. implied that *Clostridium colicanis* and *F. nucleatum* could be used as diagnosis biomarkers of gastric cancer [137]. Abed *et al*. found that Gal-GalNAC antigen was highly expressed in gastric cancer and esophageal cancer tissues, suggesting that gastric cancer and esophageal cancer could enrich *F. nucleatum* chemotaxis and affect disease progression [138].

#### Breast cancer (BC)

BC is the leading cause of cancer mortality in women and a type of cancer with different presentations among women [139]. *F. nucleatum* has recently been detected in human BC tissues and was shown to promote BC progression in a murine model [140,141]. Parhi *et al*. reported that *F. nucleatum* colonizes not only CRC, but also BC through recognition of Gal-GalNAc by Fap2 [140]. Also, metronidazole treatment can inhibit *F. nucleatum*-induced tumor exacerbation. Van der Merwe *et al*. have suggested that *F. nucleatum* may promote CRC and BC progression by activating the TLR4/ MyD88 pathway, and *F. nucleatum* also exhibits immunomodulatory effects [142]. These observations provide valuable therapeutic insights into CRC and BC: Gal/GalNAc antagonists or Fap2 antibodies could be used to inhibit the binding of *F. nucleatum* to tumours [121,140]; autophagy inhibitors, including chloroquine and hydroxychloroquine, have the potential to weaken the drug resistance of *F. nucleatum* [123,143]; PD-L1 and CD-47 antagonists would be beneficial for boosting the immune system in cancer patients [143–146].

#### Genitourinary (GU) cancers

Shuai Yuan *et al*. have summarized epidemiological studies exploring the association between PD and GU cancers, and indicated that the presence of an oral-genitourinary axis and oral microbiota may be involved in the pathogenesis of GU cancers [147,148]. Bučević Popović *et al*. are the first to report that there exists possible association of *Fusobacterium* spp. with urothelial carcinomas, at least in some bladder cancer patients [48]. They showed that *F. nucleatum* is indeed present in approximately one quarter of the tested samples by PCR-based analysis, and indicated the bacteria may play a key role in the exacerbation of the bladder cancer based on the 16S rDNA gene sequence approach. Nevertheless, more epidemiological studies and animal experiments are needed to elucidate the exact role of *F. nucleatum* in bladder cancer formation and progression. Alluri *et al*. have identified the periodontal pathogen *F. nucleatum* in prostate glands diagnosed with adenocarcinoma, but they have no evidence of whether the *F. nucleatum* found in the prostatic tissue of the individual originated from the oral cavity [149]. Meanwhile, the role of *F. nucleatum* in pathological prostate changes is unclear yet. Huang *et al*. found that there was a distinct observation of higher levels of *F. nucleatum* in cervical cancer, especially for recurrent tissues [150]. Patients with high burdens of *F. nucleatum* intratumoral infiltration exhibited correspondingly poor rates of both overall survival and progression-free survival, suggesting that *F. nucleatum* might be one potential cervical cancer diagnostic and prognostic biomarker.

### Alzheimer’s disease (AD)

AD is the most common form of dementia in older adults and refers to a central nervous system disease characterized by progressive cognitive dysfunction and memory loss [151]. In the past decade, studies have identified a relationship between periodontitis and AD, suggesting that the pathogens of periodontitis have significant implications on the development of AD [152–155].

Several putative mechanisms that could explain how periodontitis affects the central nervous system (CNS) homeostasis have been described [156], including: (i) Bacterial diffusion into the bloodstream, and once in the cerebral vessels, the inflammatory response associated with cerebrovascular atherosclerosis may induce rupture of the blood–brain barrier (BBB); (ii) Oral bacteria would migrate through the peripheral terminations of the trigeminal nerve to the trigeminal ganglion and then to the brain; (iii) Bacteria could migrate by the lymphatic circulation. While these three theories explain the possible route by which oral bacteria migrate to the brain, it has not yet been demonstrated which contributed most to the onset of AD.

With the increase of age, the permeability of the BBB increases in elderly patients. Therefore, pathogenic microorganisms could easily cross the BBB and enter the brain tissue, directly act on neurons, activate the inflammatory cascade reaction, and cause direct damage to the CNS. The bacteria entering brain tissue and their secreted virulence factors could activate a classic immune response similar in some aspects to that observed in AD, through TLR2 and TLR4 pathway, and turn inactive microglia into active ones [157,158] When activated, they could produce several inflammatory mediators such as TNF-α, IL-1β, IL-6, iNOS, and reactive oxygen species (ROS) that trigger necrosis and apoptosis of dopaminergic neurons in the CNS [159]. Additionally, oral microbial populations indirectly affect AD by secreting bacterial toxins, outer membrane vesicles (OMVs) and proinflammatory factors that flow into the brain with blood [160–163]

Earlier studies have reported that antibodies to *F. nucleatum* can be detected in the serum of patients with AD or cognitive impairment [164]. Scholars also found that the oral microbial load of *F. nucleatum* were significantly more abundant in the AD group than controls [165,166]. Recently, *in vitro* and animal experiments were conducted to preliminarily explore the pathogenesis of AD exacerbated by *F. nucleatum* [167]. Scholars reported that *F. nucleatum* LPS promoted the proliferation of microglia, which promoted the enhancement of inflammatory immune function, leading to changes in cell morphology and increased expression of inflammatory genes. *F. nucleatum* LPS could increase permeability of the BBB, and then affect the onset and development of AD. Previous studies have shown that local inflammation in the central nervous system could lead to cognitive impairment [168]. This report showed the elevations of TNF-α and IL-1β message in the brain tissue of 5XFAD mice after infection with *F. nucleatum*. In addition, in the presence of *F. nucleatum* infection, the expression of P38 protein in mouse brain tissue was significantly upregulated, as was phosphorylated P38 protein. Likewise, MyD88 protein was upregulated. *F. nucleatum* also stimulated the JNK pathway; the expression levels of phosphorylated JNK and JNK proteins were raised. Moreover, quantitative proteomics analyses were performed to detect the proteins in the brain tissues of 5XFAD mice with or without *F. nucleatum* infection [168]. The result showed that 31 proteins were obviously differentially expressed by the two groups of mice [168]. Further studies are needed to validate and explore the results of quantitative proteomics in order to find key proteins that play a significant role in signaling pathways. Furthermore, how other virulence factors of *F. nucleatum* affect the progression of AD remains unkonwn. On the whole, more efforts are required to elucidate the mechanism of *F. nucleatum* actions in AD in future studies.

### Other organ inflammation and abscesses

Han et al. have suggested that *F. nucleatum* is associated with brain, lung, liver, and splenic abscesses [34]. Recent studies also indicated that *F. nucleatum* was involved in rheumatoid arthritis (RA) and acute appendicitis [164,166,169–171]. Ebbers *et al*. firstly reported that a triple oral inoculation of pathobionts (*P. gingivalis, F. nucleatum*, and *A. actinomycetemcomintans*) combined with collagen could induce arthritis in the mouse, and oral inoculation with either *F. nucleatum* or *A. actinomycetemcomintans* alone could accelerate subsequent arthritis onset and progression [171]. Several scholars have found that *P. gingivalis, F. nucleatum* and *A. actinomycetecomitans* inhaled into respiratory tract could promote the invasion of *Pseudomonas aeruginosa* into respiratory epithelial cells and induce cytokine production and cell apoptosis [172]. *F. nucleatum* is one of the most common causative agents of Lemierre’s syndrome, a rare form of upper airways infection with a life-threatening secondary septic thrombophlebitis of internal or external jugular veins [173].

## Conclusions

**Figure 2.**
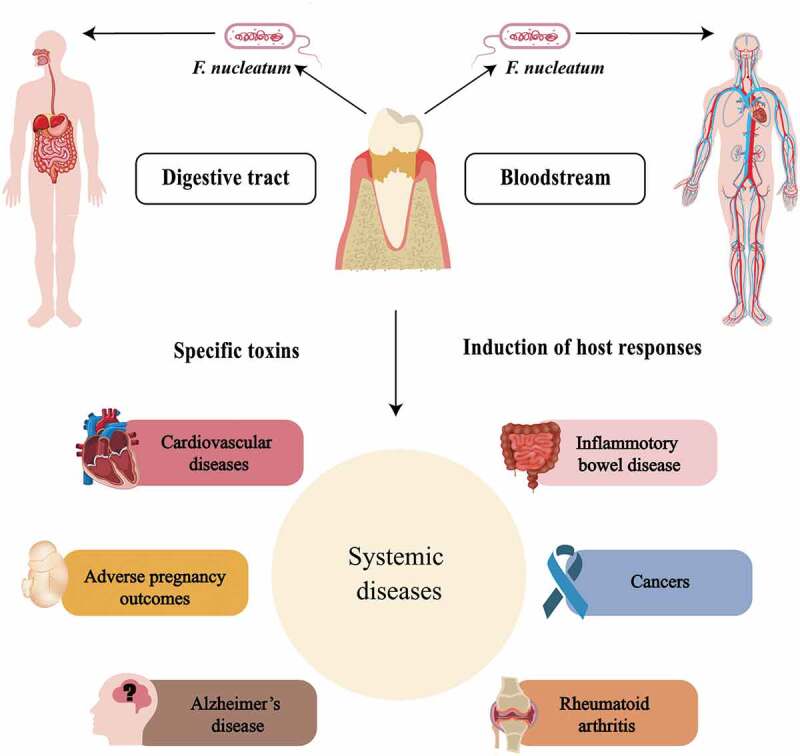
Patterns by which *F. nucleatum* invades the whole body, along with simple a schematic representation of *F. nucleatum*-associated systemic diseases.

*F. nucleatum* has been considered as an opportunistic pathogen that interacts with other microorganisms and plays a crucial role in many infectious diseases. With the development of omics technology, the occurrence and development of many diseases are closely related to the infection of *F. nucleatum*. The latest trend has been to focus on exploring possible direct and indirect links between *F. nucleatum* and certain systemic diseases, especially ACVDs and CRC, and an increasing amount of evidence has been accumulated. *F. nucleatum* not only promotes inflammation, but also binds to or invades multiple cell types, including oral, colon and placental epithelial cells, T cells, keratinocytes, and macrophages [174]. The answer to the question why *F. nucleatum* can invade the human body and influence general health lies in several key virulence mechanisms which can be broadly classified into three groups (Figure 2): (i) Colonization and invasion: *F. nucleatum* can enter the circulation and cause transient bacteremia following daily life activities, such as toothbrushing, chewing, and flossing, and after dental treatment procedures. Frequent circulation enables bacteria to access organs around the body and take part in local pathogenesis [175]. In addition, *F. nucleatum* can colonize intestinal mucosa through digestive tract [75]. (ii) Induction of host responses: *F. nucleatum* is a potent stimulator of inflammatory cytokines. Persistent local infection caused by *F. nucleatum* induces the upregulation of inflammatory cascades [38,48–50]. Chronic inflammation is believed to be the root cause of systemic disorders and is one of the leading causes of long-term health problems. Furthermore, *F. nucleatum* promotes the progression of various systemic diseases by upregulating expression of different TLRs [29,121,141], promoting macrophage M1 polarization [84], and enhancing proliferation of CD4^+^ T cell and differentiation to Th1 and Th17 [66]. (iii) Specific toxins of *F. nucleatum*, especially FadA, Fap2 and LPS, play a significant role in the induction of local diseases [15,84,114,140].

In this article, we not only summarize the adverse effects of *F. nucleatum* on multiple systemic diseases, but also discuss the impact of targeting *F. nucleatum* on the treatment of related systemic diseases. However, many of the epidemiological studies only show the correlation but not necessarily causation between *F. nucleatum* and specific diseases. Precise pathogenicity of *F. nucleatum*-related systemic diseases has yet to be uncovered, and a few conclusions are controversial. Only by continuing to investigate mechanisms involved in transformation of this oral commensal organism into systemic pathogens will we discover possible strategies for diagnostic, preventive and therapeutic purposes. Based on current research, some recommendations can be given. Firstly, it must be emphasized that oral health should be maintained as an indispensable part of a healthy lifestyle to reduce risk of bacteremia, especially for immune-compromised patients. Secondly, by investigating into the epidemiological background of *F. nucleatum* infection, high-risk groups for various related diseases can be effectively revealed, facilitating early detection and prevention among the population. Thirdly, it is of great significance for *F. nucleatum*-associated cancer patients with poor prognosis or chemotherapy-resistance to apply multi-drug combination therapy against *F. nucleatum* and employ drug administration methods similar to *Helicobacter pylori* triple therapy.
